# A dataset of necrotized cassava root cross-section images

**DOI:** 10.1016/j.dib.2020.106170

**Published:** 2020-08-12

**Authors:** Joyce Nakatumba-Nabende, Benjamin Akera, Jeremy Francis Tusubira, Solomon Nsumba, Ernest Mwebaze

**Affiliations:** a Department of Computer Science, Makerere University, Uganda; b Google AI, Ghana

**Keywords:** Cassava, CBSD, necrosis, lesions, cassava root cross-sections

## Abstract

Cassava brown streak disease is a major disease affecting cassava. Along with foliar chlorosis and stem lesions, a very common symptom of cassava brown streak disease is the development of a dry, brown corky rot within the starch bearing tuberous roots, also known as necrosis. This paper presents a dataset of curated image data of necrosis bearing roots across different cassava varieties. The dataset contains images of cassava root cross-sections based on trial harvests from Uganda and Tanzania. The images were taken using a smartphone camera. The resulting dataset consists of 10,052 images making this the largest publicly available dataset for crop root necrosis.

The data is comprehensive and contains different variations of necrosis expression including root cross-section types, number of necrosis lesions, presentation of the necrosis lesions. The dataset is important and can be used to train machine learning models which quantify the percentage of cassava root damage caused by necrosis.

**Specifications Table**

**Table:** 

Subject	Computer Vision, Machine Learning
Specific subject area	Cassava Brown Streak Disease (CBSD) Necrosis Scoring on Cassava Root Tubers
Type of data	Raw image data
How data were acquired	12-megapixel smartphone cameras
Data format	Raw data on Cassava root cross-section images are classified as either healthy or necrotic and affected by CBSD.
Parameters for data collection	Image data collected during cassava trial harvests.
Description of data collection	Images were collected in the field during cassava harvest and other images were collected in a lab setting.
Data source location	Institution: National Crop Resources Research Institute (NaCRRI), Tanzania Agricultural Research Institute (TARI) Town: Namulonge and Mwanza Country: Uganda and Tanzania
Data accessibility	Raw data is publicly available at Mendeley data. Data identification number: 10.17632/gvp7vshvnh.3 Direct URL to data: https://data.mendeley.com/datasets/gvp7vshvnh/3 The dataset citation is in Ref [1].

**Value of the Data**

1 Necrosis dataset can be used to train machine learning models which quantify the percentage of cassava root damage caused by necrosis.
2 Necrosis image data set can be used by cassava breeders as a reference and review for scores given in the field during harvest.
3 The image data set can be used for other visual experiments like cassava genetics studies which would not otherwise be possible in the field during harvest.
4 The data is comprehensive, containing all variations of necrosis that is: root cross-section types, number of necrosis lesions, presentation of the necrosis lesions.
5 This dataset is to our best knowledge the first necrosis dataset that is publicly available.

## Data description

We present an image dataset of cassava root cross-sections collected from field trials alongside agricultural experts. The data set contains healthy cassava root images and images of cassava roots affected by Cassava Brown Streak Disease (CBSD). The data was collected from the National Crop Resources Research Institute (NaCRRI) and the Tanzania Agricultural Research Institute (TARI) that hosts the national cassava breeding programs of Uganda and Tanzania respectively. The data is presented through one table and four figures. Table 1 shows a description of the dataset containing 10,052 images of cassava root cross-sections. The dataset contains both clean and necrotized cassava roots. This raw dataset is publicly available as a Mendeley repository [1].

**Table 1:** Description of the dataset from the different harvest trials in NaCRRI and TARI.

Harvest Date	Data Source	No. of Images
May 2018	NaCRRI, Uganda	4109
September 2018	NaCRRI, Uganda	251
April 2019	NaCRRI, Uganda	5055
September 2019	NaCRRI, Uganda	335
February 2020	TARI, Uganda	302
	**Total**	**10,052**

**Fig. 1:**
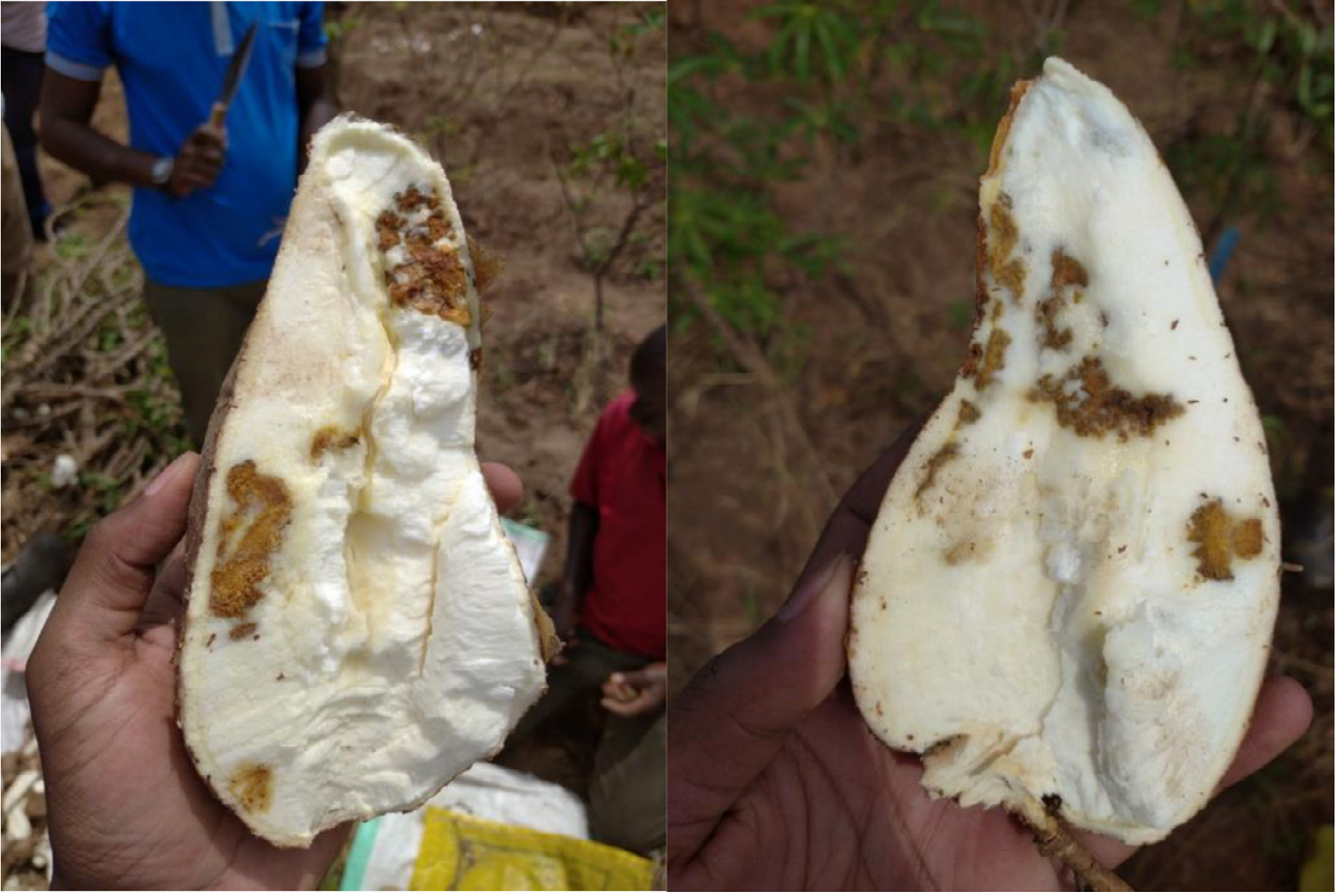
Sample images showing longitudinal cuttings of cassava roots from the trial harvest in TARI.

**Fig. 2:**
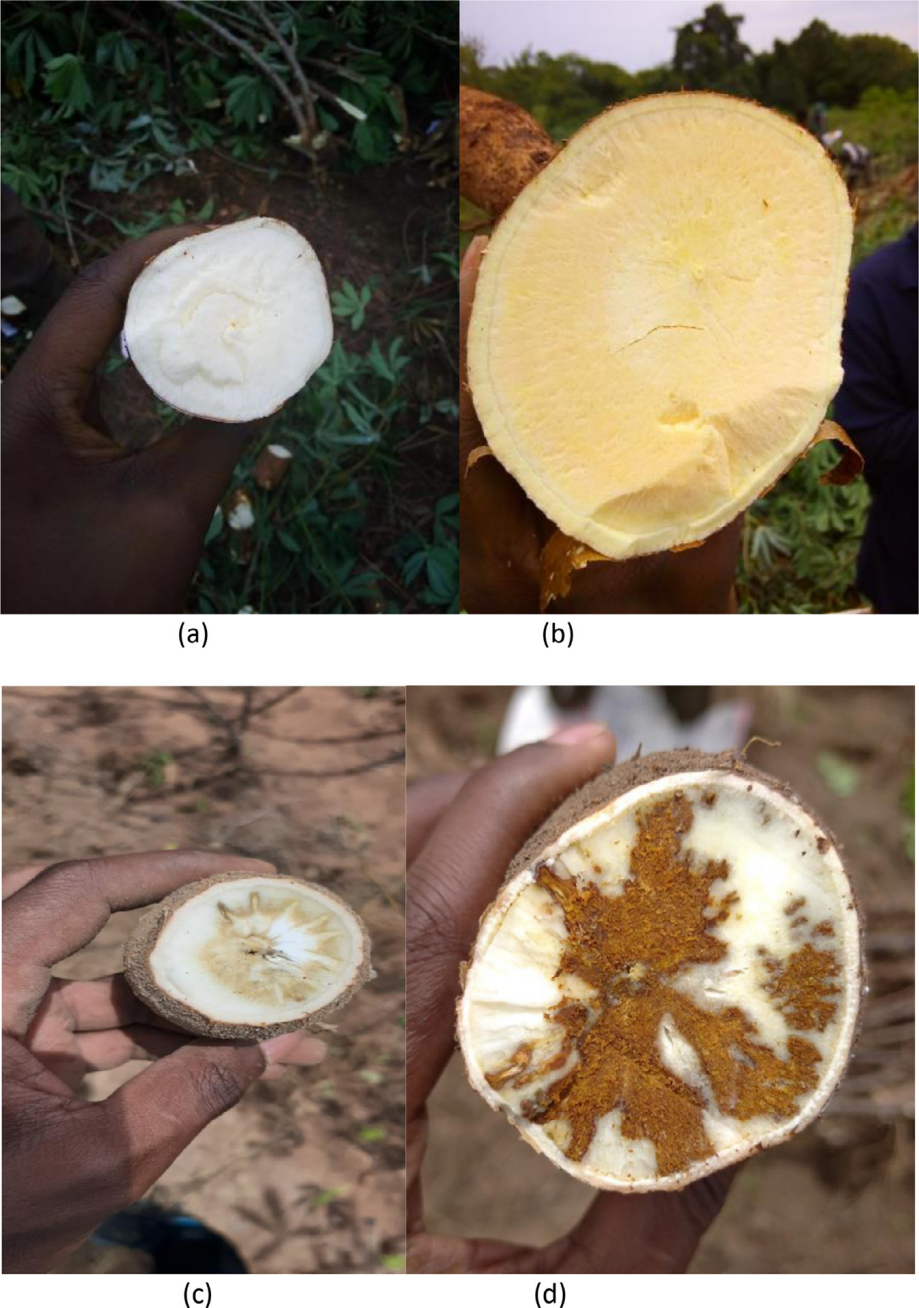
Sample images showing white fleshed (a) vs. yellow fleshed cassava (b) and white necrosis lesions (c) vs. brown necrosis lesions (d).

**Fig. 3:**
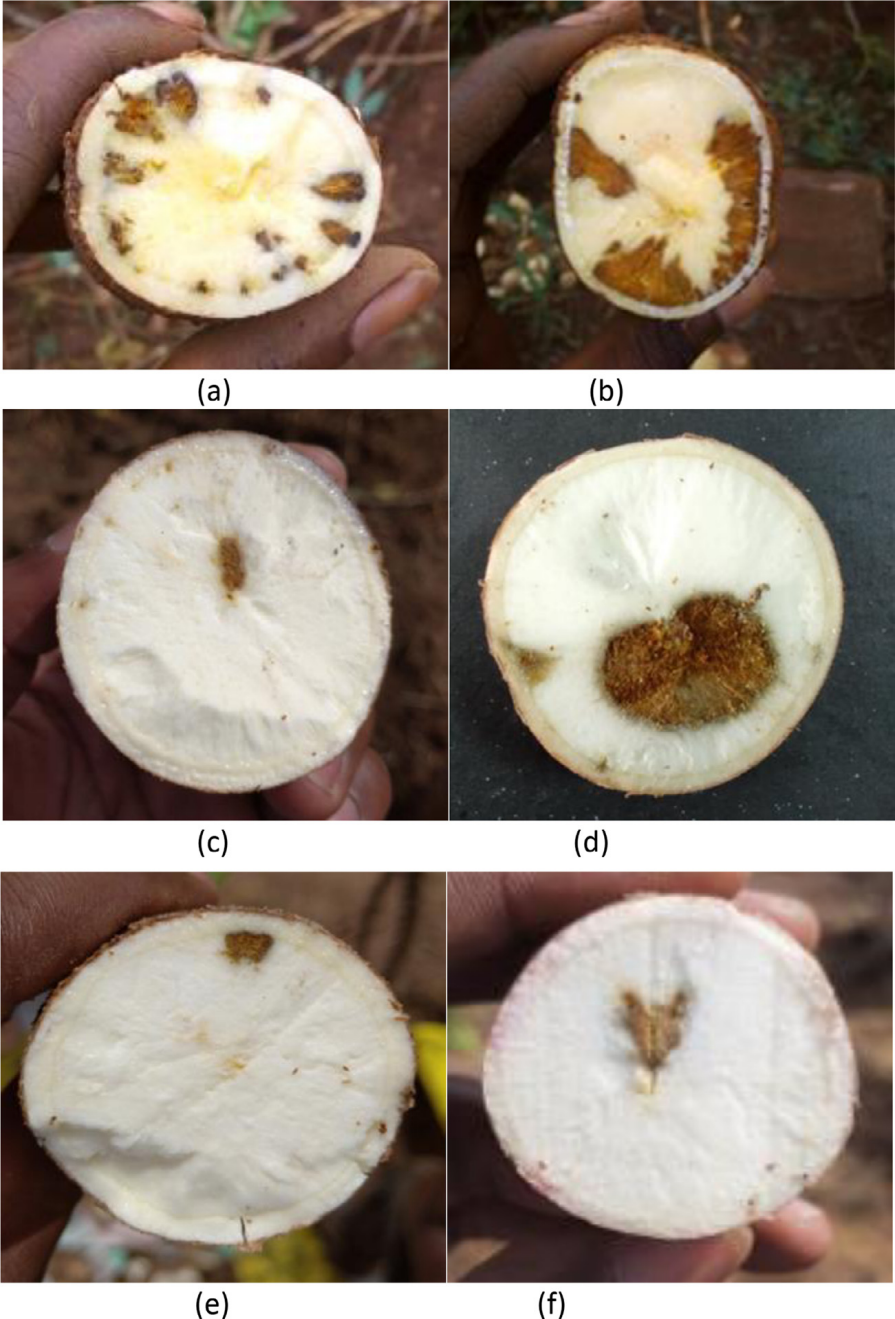
Many necrosis lesions in (a) few necrosis lesions in (b) Large necrosis lesion in (c) small necrosis lesion in (d) necrosis lesion in the center of the root in (e) and necrosis lesion at the edge of the root (f).

**Fig. 4:**
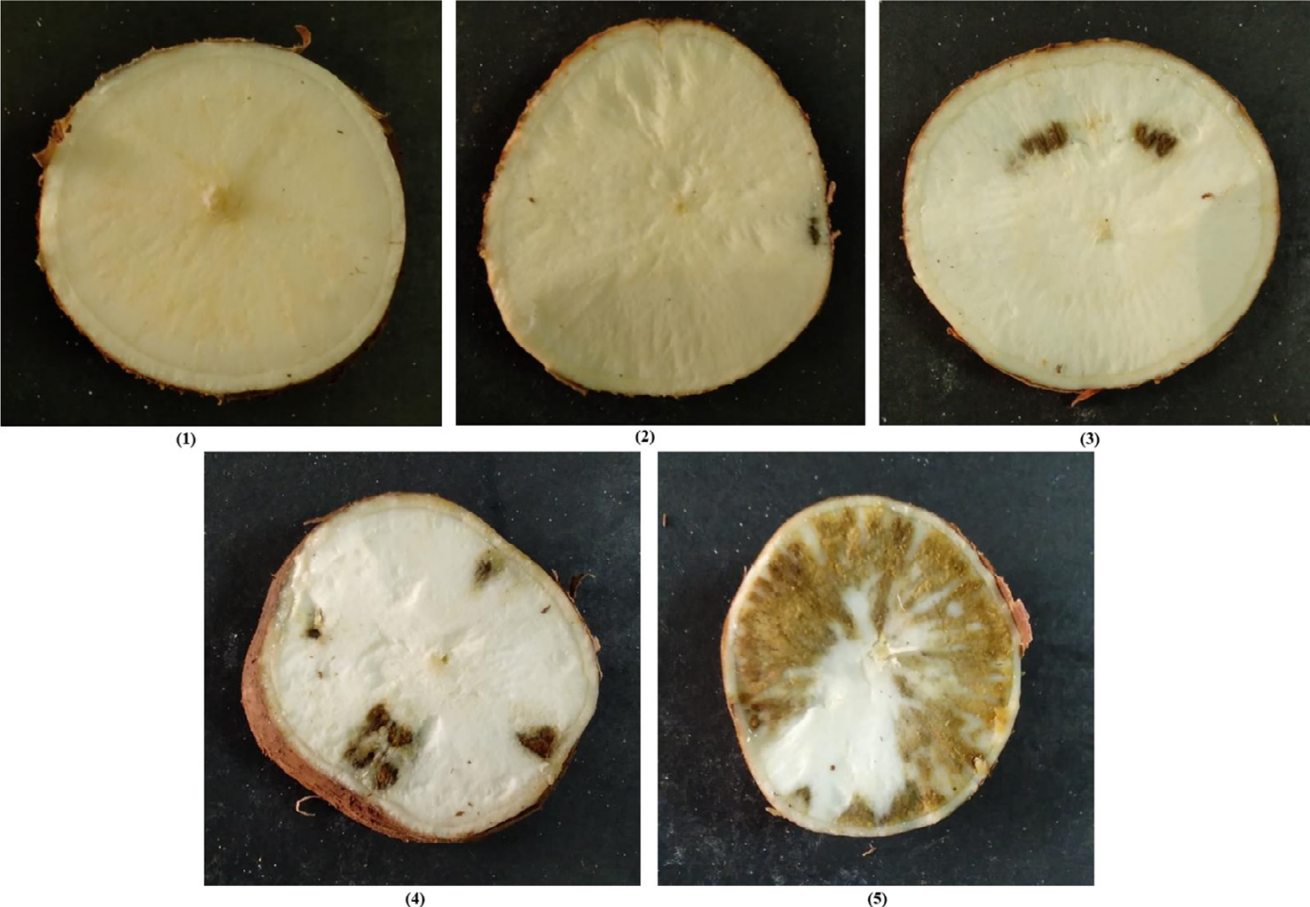
Cross-sections of cassava roots showing severity scores 1–5 for cassava brown streak disease root necrosis.

Fig. 1 shows cassava root images with horizontal slicing from a trial harvest that was carried out in TARI, Tanzania. Fig. 2 shows an image set of cassava root cross-sections. Cassava can be white fleshed (as shown in (a)) or have different variations of yellow fleshed (as shown in (b)). This figure also shows that the necrosis lesions can either be brown (c) or white (d) in color. Fig. 3 shows the manifestation of lesions on the necrotic roots. The necrosis lesions are observed to fall under three main categories: (i) number of necrotic lesions - few lesions vs. many lesions, (ii) size of the necrotic lesions - small lesions vs. large lesions, (ii) the distribution of the necrosis - lesions at the center of the root vs. lesions at the edge of the root. Finally, Fig. 4 shows an example of sample images that correspond to the severity scores on the 1–5 scale.

## 2 Experimental design, materials and methods

### 2.1 Field data collection

The dataset consists of images of cassava root cross-sections collected from NaCRRI and TARI. The cassava root cross-sections images were collected during four field trials at NaCRRI and one harvest trial in TARI as described in Table 1. The field trials were planted following the two rain seasons, i.e., April - June 2019 and September - November 2019.

Specifically, the root cross-section images collected comprised of cassava check-clones both susceptible and tolerant to CBSD root necrosis [2]. Stem cuttings used in these trials were sourced from farmers’ gardens that had *a*  ≤ 80% chance of CBSD incidence and mean severity of  ≥ 4 for shoot and or roots. Thus the CBSD infection on the cassava crops was natural and highly aided by the presence of high cassava whitefly *(Bamiscia Tabaci)* populations in the study area [3].

At harvest, 12 months after planting, the plants were uprooted and all roots were individually assessed for necrosis. This was done by slicing each root transversely 5–7 times and each time the severity of necrosis incidence was assessed using the 1–5 scale. This is a standard scale proposed by [4] and is summarized in Table 2. Images of the sliced root cross-sections were taken using a 12 megapixel smartphone camera and their corresponding quantitative scores were captured.

**Table 2:** Standard scale used by experts to correlate severity of necrosis and corresponding percentage of the extent of necrosis cover on the root.

Expert score	% of necrosis score
1	No necrosis
2	≤ 5
3	6–10
4	11–25
5	> 25

### 2.2 Data preprocessing

**Fig. 5:**
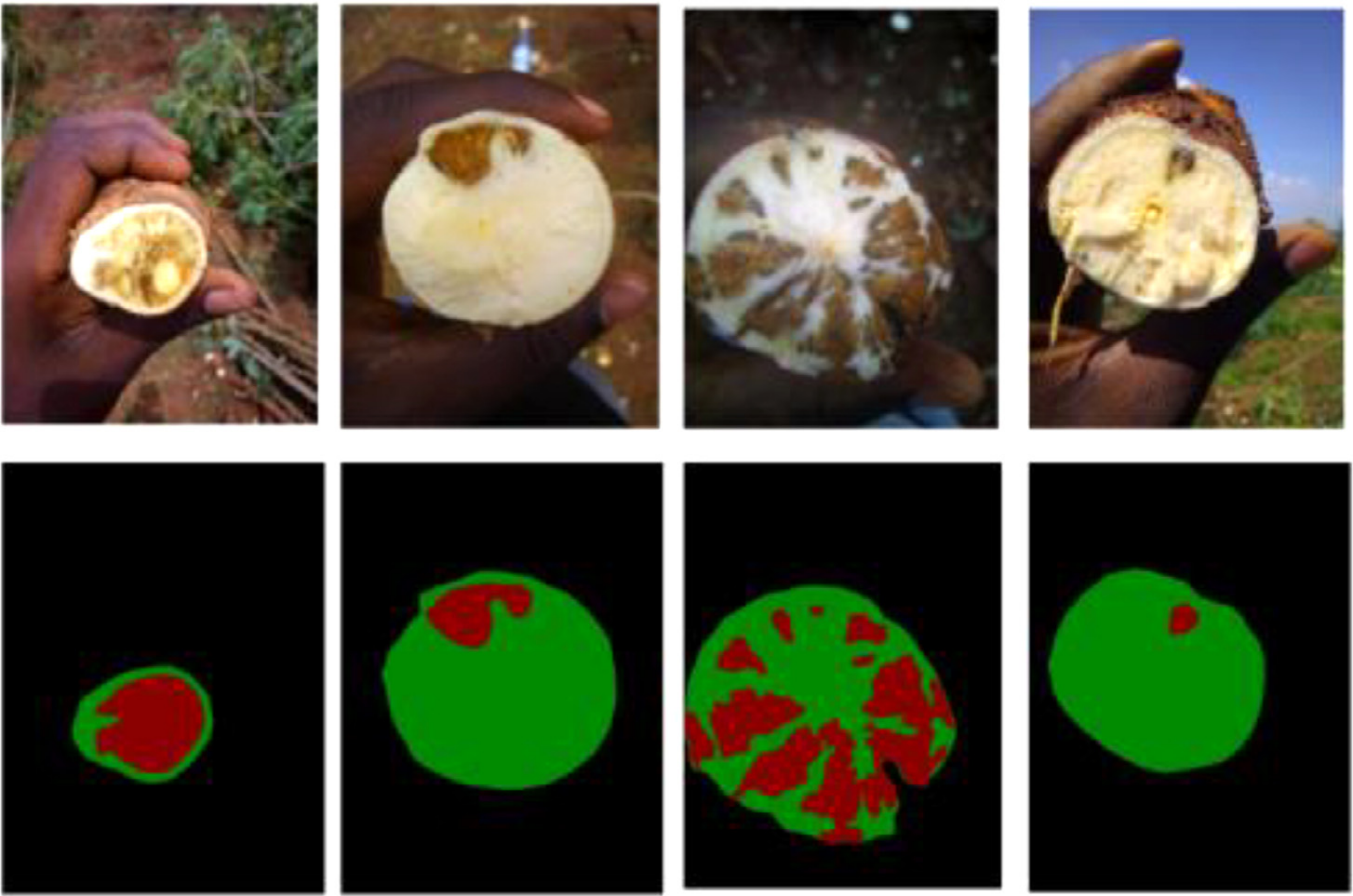
Sample input images of cassava root cross-sections and the actual masks generated from the annotation.

As discussed, the image data sets were collected during the harvest trials in the field. Since the images were collected *in-situ*, it was important to check for image quality. One of the main issues with the images was that dirt. It was important that roots did not have any dirt which would otherwise affect the score, as dirt would be considered as part of necrosis. Other issues about the images include: images were not blurred, they were in focus, images did not consist of random objects accidentally and that the distance from the camera that was sufficient enough not to affect the quality of the images. A sample of 1036 images were annotated by a group of two volunteers in the Makerere Artificial Intelligence Lab. The annotation was performed using the open source tool *LabelMe*.^1^ This task involved manually tracing the root lesions resulting into a colored mask showing different regions of the image. The masks are segmented to show three areas in each image: the root (shown as green), necrosis (shown as red) and everything else, i.e., background (shown as black) as seen in Fig. 5. The groundtruth percentages for necrosis can be calculated using the groundtruth masks.

## Credit author statement

Joyce Nakatumba-Nabende: Conceptualization, Methodology and Writing. Benjamin Akera: Writing - Original Draft, Jeremy Francis Tusubira: Data curation, Reviewing, Editing, Solomon Nsumba, Ernest Mwebaze: Reviewing and Editing.

## Declaration of Competing Interest

The authors declare that they have no known competing financial interests or personal relationships that could have appeared to influence the work reported in this paper.

## Appendix A Supplementary Data

• The raw dataset described in this paper is hosted on this url: https://data.mendeley.com/datasets/gvp7vshvnh/3

## Appendix B Supplementary materials

Image, application 1

## Footnotes

1 https://github.com/wkentaro/labelme
